# Characterization of Dark Septate Endophytes Under Drought and Rehydration and Their Compensatory Mechanisms in *Astragalus membranaceus*

**DOI:** 10.3390/microorganisms12112254

**Published:** 2024-11-07

**Authors:** Yali Xie, Xueli He, Duo Wang, Menghui Wang, Wanyun Li, Wenjing Chen, Xianen Li, Chao He

**Affiliations:** 1 Institute of Medicinal Plant Development, Chinese Academy of Medical Sciences & Peking Union Medical College, Beijing 100193, China; xieyali998@163.com (Y.X.); liwanyun0623@126.com (W.L.); 2 School of Life Sciences, Hebei University, Baoding 071002, China; xlh3615@126.com (X.H.); wd18740746653@163.com (D.W.); wangmenghui0225@163.com (M.W.); chenwenjing09@163.com (W.C.)

**Keywords:** drought stress, rehydration, dark septate endophytes, physiology, untargeted metabolites, *Astragalus membranaceus*

## Abstract

Drought is the most significant abiotic stress that impedes agroforestry development. In nature, drought tolerance also depends on the ability to compensate after water restoration. Dark septate endophytes (DSEs) are believed to enhance plant tolerance in drought environments. However, the compensatory mechanisms of DSEs for rehydration after drought stress have not been reported. To assess the drought tolerance and compensatory capacity of DSEs, the following DSEs were investigated in this study using solid–liquid screening and potting tests under different drought gradients, rehydration conditions, and field water-holding capacities: *Stagonosporopsis lupini*, *Microsphaeropsis cytisi*, *Macrophomina pseudophaseolina*, *Paraphoma radicina*, *Alternaria alstroemeriae*, *Alternaria tellustris*, and *Papulaspora equi*. The results showed that *M. pseudophaseolina* reached the maximum diameter for plate growth in only 4 d. In a liquid shaker, the biomass of *S. lupini* peaked after rehydration. The Mantel heatmap indicated that lipid metabolites were significantly expressed in *M. pseudophaseolina* and *S. lupini* under drought stress. Correlations between drought tolerance indexes and amino acid metabolites increased dramatically in both DSEs after rehydration. Moreover, in rehydration after drought, the treatments inoculated with *M. pseudophaseolina* and *S. lupini* showed significant increases in root weight of 20.36% and 23.82%, respectively, compared with the uninoculated treatment.

## 1. Introduction

As global climate change intensifies, drought has emerged as the primary abiotic stressor hindering the advancement of agroforestry and ecological restoration [1]. Plants can alleviate or counteract the impacts of drought stress by altering their physical structure and biological processes [2]. In nature, the effects of environmental changes on plants are dynamic and alternating. Plants’ drought tolerance is demonstrated by their capacity to recover and compensate after rehydration, as well as their adaptation to drought stress [3]. Studies have shown that rehydration after a drought usually activates the activity of soil microorganisms to some extent and stimulates compensatory effects in plants [4]. The current study primarily focuses on rehydration after mild or moderate drought stress, during which plant growth and yield undergo overcompensation or compensatory responses. Conversely, severe drought stress can inhibit plant growth and physiological activities, making it more difficult to induce compensatory effects after rehydration [5]. For example, it has been demonstrated that after experiencing prolonged mild drought and rehydration, ectomycorrhizal fungi increase their seedling root uptake area, improve their seedling root vigor, and exhibit some compensatory effects [6]. After rehydration, Ericoid mycorrhizas (ERM) fungi can enhance the drought tolerance and restoration of *Rhododendron annae* [7]. In a study on the inoculation of ectomycorrhizal fungi on *Pinus massoniana* seedlings, it was discovered that the fungi could enhance the growth and development of *Pinus massoniana* seedlings when water was reintroduced after mild and moderate drought stress. However, it was challenging to recover the growth index of the seedlings when water was reintroduced after severe drought stress [8]. Therefore, the adaptation of plants to adversity was closely related to the presence of microorganisms. The utilization of beneficial fungi to enhance plant adaptation to drought stress and compensation after water recovery is an urgent issue.

*Astragalus membranaceus* is a medicinal plant that is widely distributed in the desert steppe and arid inland regions of northwest China. The medicinal value of *A. membranaceus* was first recorded in the Shennong Herbal Classic. As a rare medicinal material that is commonly used in China, *A. membranaceus* clears heat and drying dampness, strengthens the spleen, increases vitality, detoxifies, and reduces swelling [9]. However, with the increase in domestic and foreign demand, the degradation of vegetation, and the destruction of habitats caused by continuous mining, wild *A. membranaceus* resources are scarce. At present, artificial cultivation of *A. membranaceus* has become the main source for the market, but studies have found that although the planting environment of cultivated *A. membranaceus* is better than that of wild *A. membranaceus*, the quality of cultivated *A. membranaceus* is unstable, and the content of medicinal ingredients is far lower than that of wild *A. membranaceus* due to the mixed germplasm sources of cultivated *A. membranaceus* and the non-standard planting methods [10]. Therefore, it is urgent to improve the yield and quality of cultivated *A. membranaceus*. Studies have suggested that utilizing beneficial microorganisms such as dark septate endophytes (DSEs) to improve plant growth and tolerance in arid environments is considered an effective approach [11].

DSEs are mostly ascomycetes, and they can infect plant roots and form typical dark septate hyphae and microsclerotic structures. In harsh environments, these fungi can improve plant performance and stress resistance [12]. Prior research indicated that DSEs can establish a complex network within plant roots, stimulate enzyme and hormone secretion, bolster plant resilience by modulating superoxide dismutase (SOD) activity, glutathione (GSH), and soluble protein concentrations, and safeguard plants against biotic and abiotic stressors [13]. The findings demonstrated that the relationship between DSEs and host plants is essential for plant survival in severe settings [14]. However, little is known about how DSEs can tolerate extreme drought and about their compensation mechanism after rehydration.

When plants are subjected to drought stress, they release more reactive oxygen species (ROSs). To lessen or offset the harmful effects of ROSs, plants either create or activate a range of metabolites that have antioxidant activity [15]. Research indicates that variations in the types and amounts of metabolites generated by plants can signify their adaptability to environmental conditions [16]. Transcriptomic and metabolomic analyses revealed that drought-treated *Illicium bifengpi* recovered most physiological and metabolic indicators after rehydration [17]. Rehydration relieves soil drought conditions and subsequently improves the metabolism of microorganisms, such as fungi, making them better able to resist environmental stresses [4]. However, few studies have shown how metabolites accumulate in DSEs under drought and rehydration conditions. In this study, seven DSE strains were used to investigate the response of the growth and metabolic processes of DSE strains to a PEG concentration gradient that was set up to simulate rehydration tests after severe drought. Meanwhile, the effect of DSE inoculation on the growth of *A. membranaceus* under different soil moisture content and rehydration conditions was investigated through pot experiments. We propose the following research questions: (1) Can DSEs tolerate different drought stresses? (2) Does rehydration of DSEs after severe drought stress have a compensatory effect? (3) Can rehydration enhance the growth performance of the *A. membranaceus*-DSE symbiosis? These data help establish a research system for utilizing microorganisms to assist in plants’ drought resistance and improve their water use efficiency, providing a basis for the water-saving cultivation of crops.

## 2. Materials and Methods

### 2.1. Fungal Materials

This research utilized seven DSE strains obtained from the roots of various plants in arid regions as experimental subjects (Table 1). Ribosomal RNA partial sequence numbers of these DSEs were uploaded to GenBank, and the strains are listed below: *Stagonosporopsis lupini* (OP363602, *Sl*), *Microsphaeropsis cytisi* (ON413886, *Mc*), *Macrophomina pseudophaseolina* (MZ506881, *Mp*), *Paraphoma radicina* (MT723853, *Pr*), *Alternaria alstroemeriae* (MZ505449, *Aa*), *Alternaria tellustris* (MW548078, *At*), and *Papulaspora equi* (MW548086, *Pe*). Each strain was cultured for 14 d before follow-up experiments. They were preserved at 4 °C in the Mycorrhizal Biology Laboratory at Hebei University.

### 2.2. Growth Conditions

Seeds of *A. membranaceus* were procured from Gansu Province, China, and preserved at 4 °C. Evenly full *A. membranaceus* seeds were selected, rinsed with distilled water three times, soaked in distilled water for 12 h, put into a seedling bowl with a small amount of distilled water, and put into an incubator at 25 °C. Seeds were removed from the seedling pots after about a week of germination. The growth substrate used was a mixture of 1:2 (*W*:*W*) sand (less than 2 mm) and soil. The experiment was designed as a completely randomized two-factor (4 DSE inoculation treatments × 5 drought stress treatments) block experiment. Factor 1 was the DSE strains with inoculated *Mp* and *Sl* strains and a blank control group. Factor 2 was a soil moisture treatment. Soil moisture content was maintained at 70%, 50%, and 30% of the field water-holding capacity by using the weighing method; these were set as the normal moisture, mild drought, and severe drought levels, respectively. The 50% and 30% drought treatments were each performed with eight replicates, and the 70% treatment was performed with four replicates. The 50% and 30% drought treatments were each rewatered to the normal moisture level of 70% after 45 d of drought treatment (Table 2). These treatments were designed to assess the plant response to different water supplies and after rehydration.

A total of 800 g of mixed soil–sand substrate was placed in plastic pots (13 cm caliber, 10 cm bottom diameter, 12 cm height). Fungal inocula that were 7 mm in diameter were picked from the PDA medium; four inocula were placed on the top layer of each pot’s substrate and, finally, covered with 550 g of the mixed soil–sand substrate. Four blank PDA media were obtained using the same method as DSE inoculation [18]. Four seedlings with good and uniform growth were selected and planted in plastic pots within an artificial climate incubator with a light cycle of 14/10 h and 24/22 °C (day/night). The experiment was started on 24 February 2024, and harvested on 24 June 2024, after 4 months. After 45 d, *A*. *membranaceus* was subjected to different drought treatments, and the different drought treatments were rewatered to 70% soil moisture content after continuing the incubation for 45 d. The plants were harvested 1 month after rewatering. Water loss was periodically replenished with distilled water during the experimental period, and the soil water content was weighed to maintain the soil water content at the corresponding moisture condition. The incubator’s position was randomly altered weekly to prevent positional factors from influencing the results. The height of aboveground plants and the number of leaves were recorded, and the chlorophyll content of the plants was determined using a portable chlorophyll meter (SPAD-502 plus., Konica Minolta Inc., Tokyo, Japan) before harvesting. The roots of *A*. *membranaceus* plants below ground were harvested, rinsed with water to remove residual soil, blotted with absorbent paper, and their fresh weight was recorded.

### 2.3. Determination of DSE Drought Tolerance in Solid Plate Culture

A total of 120 mL of PDA medium was prepared, and 6.32, 13.33, 21.18, 30, and 40 g of polyethylene glycol (PEG-6000) and 0.7, 0.9, 1.2, 1.7, and 2.2 g of phytol coagulant were used to modify the osmotic pressure of the medium to simulate a drought-stressed environment. This resulted in PEG-6000 concentrations of 5%, 10%, 15%, 20%, and 25% (PDA medium was obtained from Beijing Aoboxing Bio-Technology Co., Ltd., Beijing, China) [19]. After 14 d of incubation, DSE colony inocula that were 5 mm in diameter were obtained from the peripheral region and subsequently placed in the center of the PDA medium with various PEG-6000 drought stress gradients. Each treatment was replicated three times and incubated for 14 d in the dark within an inverted incubator set at 27 °C in a thermostat.

### 2.4. Determination of the Drought Tolerance of DSE in Liquid Culture

To simulate different drought stress gradients in liquid culture, each 250 mL conical flask held 120 mL of potato dextrose in water (PD) as a liquid medium, which was then supplemented with 0, 6.32, 13.33, 21.18, 30, and 40 g of PEG-6000 solids to achieve concentrations of 0, 5, 10, 15, 20, and 25% of PEG-6000, respectively (the liquid PD medium was sourced from Qingdao Hope Bio-Technology Co., Ltd., Qingdao, China) [8]. After 14 d of activation, three 5 mm inocula were removed from the DSE colonies and transferred into a liquid PD medium with different drought concentrations. Each treatment was repeated three times. Cultures were incubated on a constant-temperature shaker (27 °C, 150 r/min) for 14 d. After incubation in a liquid medium, the culture solution and mycelium were separated using an SHD-III multipurpose circulating water vacuum pump (Henan Tester Instrument Co., Ltd. Zhengzhou, Henan, China).

### 2.5. Determining Post-Drought Rehydration Compensation for DSEs Under Liquid Culture Conditions

The mycelium and culture medium were separated using an SHD-III multipurpose circulating water vacuum pump after culturing with liquid PD medium treated with 25% drought on a constant-temperature shaker (27 °C, 150 r/min) for 14 d (Suzhou Jiemei Electronic Co., Ltd. Suzhou, Jiangsu, China). Subsequently, the separated mycelium was inoculated into a liquid PD medium without any drought concentration (0%). The culture was further incubated in a constant-temperature shaker (27 °C, 150 r/min) for 7 d until the 21st day to simulate rehydration after a drought. After 21 d, an SHD-III multipurpose circulating water vacuum pump was used to separate the mycelium and culture medium in the 0%, 25% drought, and rehydration treatments.

### 2.6. Determination of the Physiological Indicators of DSEs in Drought and Rehydration Treatments

Two segments of the fresh mycelia were randomly separated. A fraction was employed to assess the concentrations of melanin, malondialdehyde (MDA), glutathione (GSH), and soluble protein, as well as the activities of catalase (CAT), peroxidase (POD), and superoxide dismutase (SOD). In order to determine the content of soluble sugar, part of the mycelium was dried at 80 °C to a constant weight. The dry weight was recorded, and the total biomass of the DSE strain was calculated by converting the proportion of water content of the partial mycelium as follows: total biomass = total fresh weight × dry weight/partial fresh weight. The pH of the culture broth was measured using a pH meter (Spectrum Technologies, Inc. Haltom City, TX, USA).

NaOH extraction was used to measure the amount of mycelial melanin [20]. Initially, 0.05 g of fresh mycelium was weighed using an electronic scale, then combined with 1 mol L^−1^ sodium hydroxide, heated for 5 h at 100 °C, and then centrifuged for 15 min at 10,000× *g*. The absorbance value was measured at 400 nm with a spectrophotometer.

The mycelial MDA content was determined using the thiobarbituric acid (TBA) method [21]. First, 0.2 g of fresh mycelium was weighed in a 5 mL centrifuge tube using an electronic balance. Then, 4 mL of 10% trichloroacetic acid was added, and the mixture was ground evenly with a high-throughput tissue grinder (Ningbo Scientz Biotechnology Co., Ltd. Ningbo, China). The homogenate was then centrifuged at 10,000× *g* for 10 min. Subsequently, 2 mL of supernatant was absorbed in a glass test tube, and 2 mL of 0.5% thiobarbituric acid (TBA) was added to a boiling water bath for 20 min. The mixture was rapidly chilled. A spectrophotometer was employed to measure the absorbance at wavelengths of 450, 532, and 600 nm.

The concentration of GSH in the mycelium was determined using the 5, 5-dithiobis (2-nitrobenzoic acid) (DTNB) method [22]. Initially, 0.2 g of fresh mycelium was weighed in a 5 mL centrifuge tube using an electronic balance. Subsequently, 4 mL of 10% trichloroacetic acid was added, pulverized using a high-throughput tissue grinder, and centrifuged at 10,000× *g* for 10 min. Then, 0.25 mL of the supernatant was aspirated into a glass test tube, and 2.6 mL of 150 mmol L^−1^ NaH_2_PO_4_ and 0.15 mL of DTNB were added in turn, mixed, and allowed to react at 30 °C for 10 min. The absorbance was then measured using a spectrophotometer at a wavelength of 412 nm.

The Thomas Brilliant Blue method was employed to ascertain the soluble protein content [23]. Initially, 0.2 g of fresh mycelium was weighed in a 5 mL centrifuge tube using an electronic balance. Subsequently, 4 mL of 50 mM phosphate buffer (pH 7.8) was added, and the mixture was ground with a high-throughput tissue grinder. The mixture was then centrifuged at 10,000× *g* for 15 min. In a glass test tube, absorb 0.1 mL of the supernatant. Subsequently, 0.9 mL of distilled water and 5 mL of Thomas Brilliant Blue were added, mixed, and allowed to stand for 5 min. A spectrophotometer was employed to measure the absorbance at 595 nm.

Anthrone colorimetry was employed to ascertain the soluble sugar content of mycelium [24]. Initially, 0.05 g of desiccated mycelium was weighed in a 5 mL centrifuge tube using an electronic balance. Following the grinding of the sample, 4 mL of 80% alcohol was added. The material underwent two extractions with 80% ethanol (*v*/*v*) at 80 °C, and the supernatant was thereafter transferred to a 10 mL centrifuge tube. Afterward, a small quantity of activated charcoal was introduced, and a water bath was maintained at 80 °C for 30 min. The supernatant was diluted with distilled water to a volume of 10 mL. Then, 0.25 mL of the filtrate was strained and filtered into a glass tube. Subsequently, 5 mL of anthrone reagent was added to the tube, and the mixture was placed in a boiling water bath for 10 min. A spectrophotometer was employed to measure the absorbance at a wavelength of 625 nm.

The UV absorption method was used to measure the mycelium’s CAT activity [25]. To the ice bath, 0.5 mL of 50 mM phosphate buffer (pH 7.8) was added after 0.1 g of fresh mycelium was weighed using an electronic balance in a 5 mL centrifuge tube. After being ground using a high-throughput tissue grinder, the samples were centrifuged for 15 min at 4 °C at 15,000× *g*. The 3 mL reaction system was then filled with 1 mL of 0.3% H_2_O_2_ and 1.95 mL of water (grade 1). To start the process, 0.05 mL of enzyme extract was added at the end. It was given a good shake and timed right away. A unit of CAT enzyme activity of 0.01 was used to evaluate the rate of absorbance (OD value) decline at 240 nm.

Guaiacol colorimetry was employed to determine the POD activity of the mycelium [26]. Initially, 0.1 g of fresh mycelium was weighed in a 5 mL centrifuge tube using an electronic balance. Subsequently, 0.5 mL of 50 mM phosphate buffer (pH 7.8) was added to the ice bath. The sample was subsequently ground using a high-throughput tissue processor, and the homogenate was centrifuged at a rotational speed of 15,000× *g* and 4 °C for 15 min. The 3 mL reaction system was then filled with 1 mL of 0.3% H_2_O_2_, 0.95 mL of 0.2% guaiacol, and 1 mL of 50 mM phosphate buffer (pH 7.8). The reaction was initiated by the addition of 0.05 mL of enzyme solution at the end. The rate of increase in absorbance (OD value) was measured at 470 nm, where 0.01 was considered one unit of peroxidase (PODase) activity.

The nitrotetrazolium chloride blue reduction method was employed to ascertain the activity of SOD in the mycelia [27]. After weighing 0.2 g of fresh mycelium using an electronic balance in a 5 mL centrifuge tube, 4 mL of 50 mM phosphate buffer (pH 7.8) was added to an ice bath. The sample was subsequently centrifuged for 15 min at a speed of 10,000× *g* and 4 °C after being ground in a high-throughput tissue grinder. Subsequently, 0.3 mL of supernatant was absorbed, and 3.8 mL of 50 mM phosphate buffer (pH 7.8), 0.3 mL of L-methionine, 0.3 mL of nitro-tetrazolium blue chloride, and 0.3 mL of riboflavin were added in a sequential manner. The sample was incubated in a light incubator for 20 min after mixing, and the absorbance was measured at a wavelength of 560 nm using a spectrophotometer.

### 2.7. DSE Metabolic Secretion Analysis

Mycelium cultured for 21 d was separated from the culture broth using an SHD-III multifunctional recirculating water vacuum pump to collect the secretions of *Sl* and *Mp* (0%, 25%, and rehydration). The isolated medium was transferred to a 10 mL centrifuge tube, and all samples were stored at −80 °C for sequencing analysis (Shanghai Majorbio Bio-pharm Technology Co., Ltd., Shanghai, China). The raw data were uploaded to the Metabolights database with the login number MTBLS10269. The precise techniques of collection and extraction were as follows: all materials were first freeze-dried, and 20 mg were then placed in a 2 mL centrifuge tube for grinding using a 6-mm-diameter grinding bead. For the extraction treatment, 200 µL of the extraction solution (methanol: water = 4:1 (*v*:*v*)) was then added. Ultimately, the sample was centrifuged for 30 min at −20 °C, and the supernatant was removed for detection using liquid mass spectrometry (Table S1). Throughout the run, quality control (QC) samples were added to the samples in order to monitor and evaluate the stability of the system and the precision of the experimental findings. The collected raw data were imported into Progenesis QI v3.0 (Waters Corporation, Milford, CT, USA) for processing, baseline filtering, peak identification, integration, retention time correction, peak alignment, etc.; subsequently, the data were statistically analyzed using principal component analysis (PCA) and difference volcano plots to screen for VIP > 1 and *p* < 0.05 of differential metabolites. The mass-to-charge ratio (*m*/*z*), retention time, and peak area data tables were gathered from the HMDB (http://www.hmdb.ca/, accessed on 6 July 2024) and Metlin (https://metlin.scripps.edu/, accessed on 6 July 2024) databases to create data matrices.

### 2.8. Statistical Analysis

In this investigation, the effects of drought and rehydration conditions on the antioxidant enzyme activities, melanin, MDA, osmoregulatory substances, and biomass of DSE strains were assessed using a two-factor analysis of variance (ANOVA). Statistical analysis and significant difference analysis (*p* < 0.05) were conducted using the SPSS 26.0 software and the Duncan test. Origin Version 2021 software was utilized to create a bar chart. The “vegan” software utility in RStudio version 4.3.2 was employed to conduct variance decomposition analysis (VPA) to investigate the impact of drought and rehydration treatment on DSE growth indicators. Using the R software package Ropls version 1.6.2, we conducted PCA and differential metabolite analyses. The SciPy (Python) software Version 1.0.0 tool was utilized to perform hierarchical clustering, correlation analysis, and KEGG pathway enrichment analysis of metabolites across different treatments [28]. Furthermore, Mantel correlation heatmaps were employed to illustrate the correlations between DSE growth, physiological indexes, and metabolism in response to drought and rehydration treatments (https://www.chiplot.online/, accessed on 16 September 2024).

## 3. Results

### 3.1. Effects of Drought Stress on the Morphology and Growth of DSE Colonies

**Figure 1:**
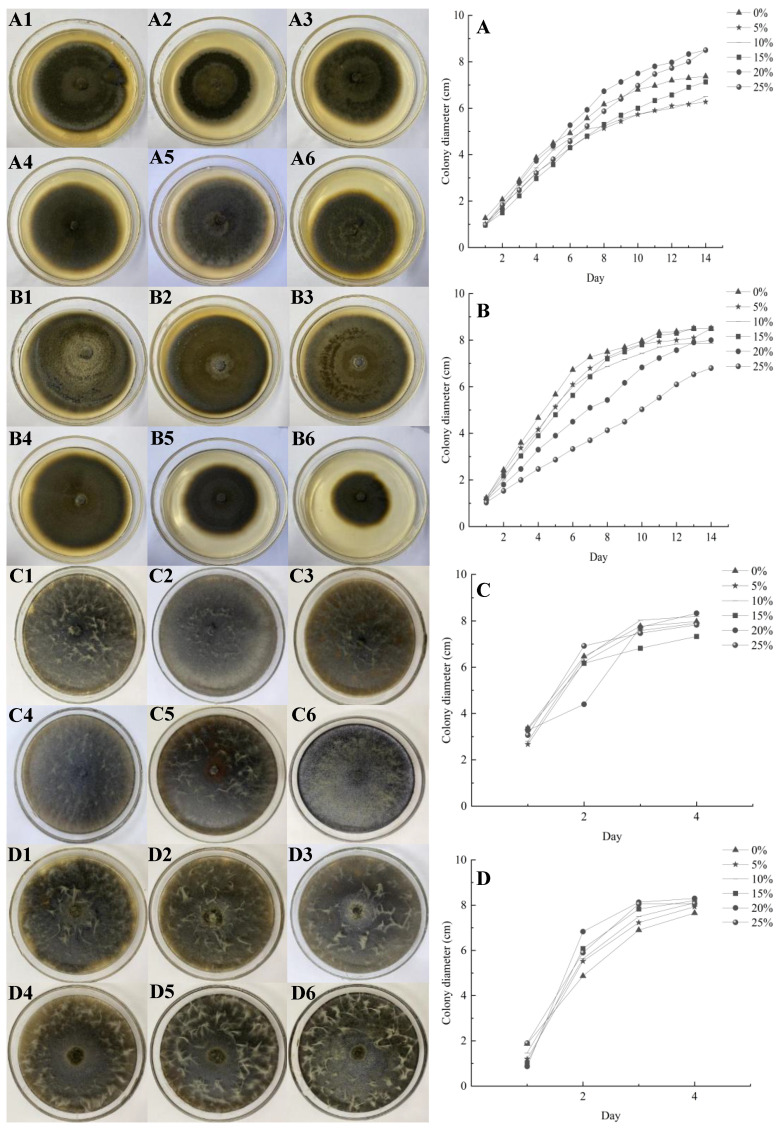

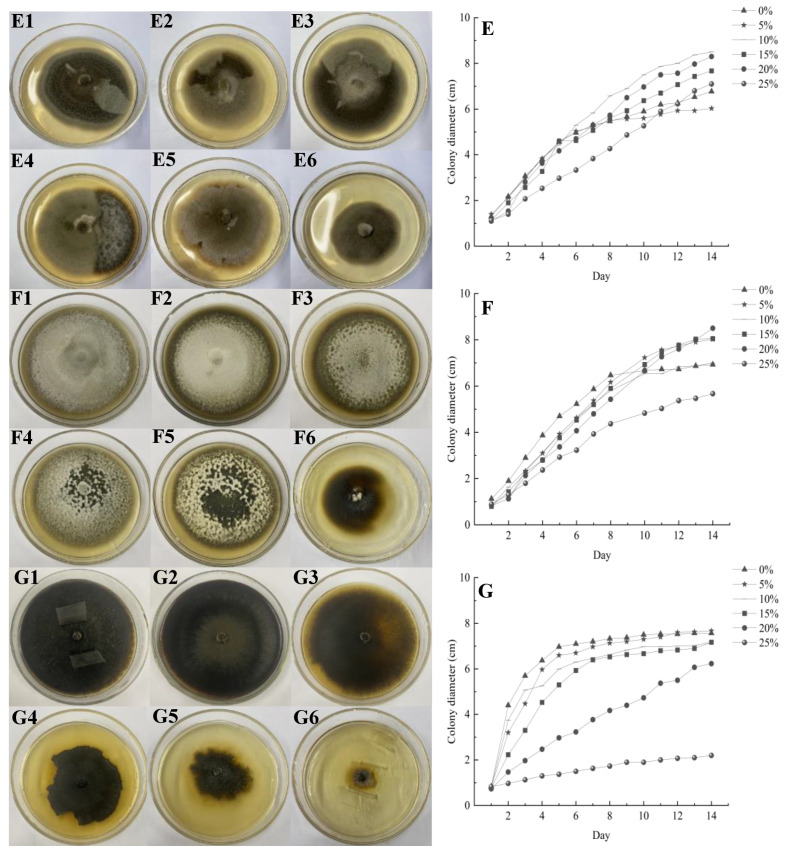
Morphological map and growth curves of DSE colonies under different drought stresses. (**A**–**G**) denote *S. lupini*, *M. cytisi*, *M. pseudophaseolina*, *P. radicina*, *A. alstroemeriae*, *A. tellustris,* and *P. equi*, respectively. Numbers 1–6 indicate drought stress gradients of 0, 5, 10, 15, 20, and 25%, respectively.

All strains, except the *Pe* strain, thrived on drought-stressed plates as the concentration of PEG-6000 increased (Figure 1A–G). Various DSEs exhibited distinct growth patterns under different levels of drought stress. The growth rate of the *Sl* strain increased with the increase in the drought stress gradient on the 14th day, and the 20% PEG-6000 concentration was the first to attain the maximum plate growth diameter. *At* the 25% concentration of PEG-6000, the *Aa* strain exhibited a faster growth rate than the *At* strain. The maximum growth rate of *Mc* was observed at a concentration of 15% PEG-6000. The *Mp* and *Pr* strains achieved maximum plate growth diameters at a PEG-6000 concentration of 20% on day 4, suggesting that higher levels of drought stress led to an acceleration in growth rates. The huge growth advantage of DSEs in the plate drought experiment was evident.

### 3.2. Effects of Different Drought Stress Treatments on the Tolerance of DSEs

**Figure 2:**
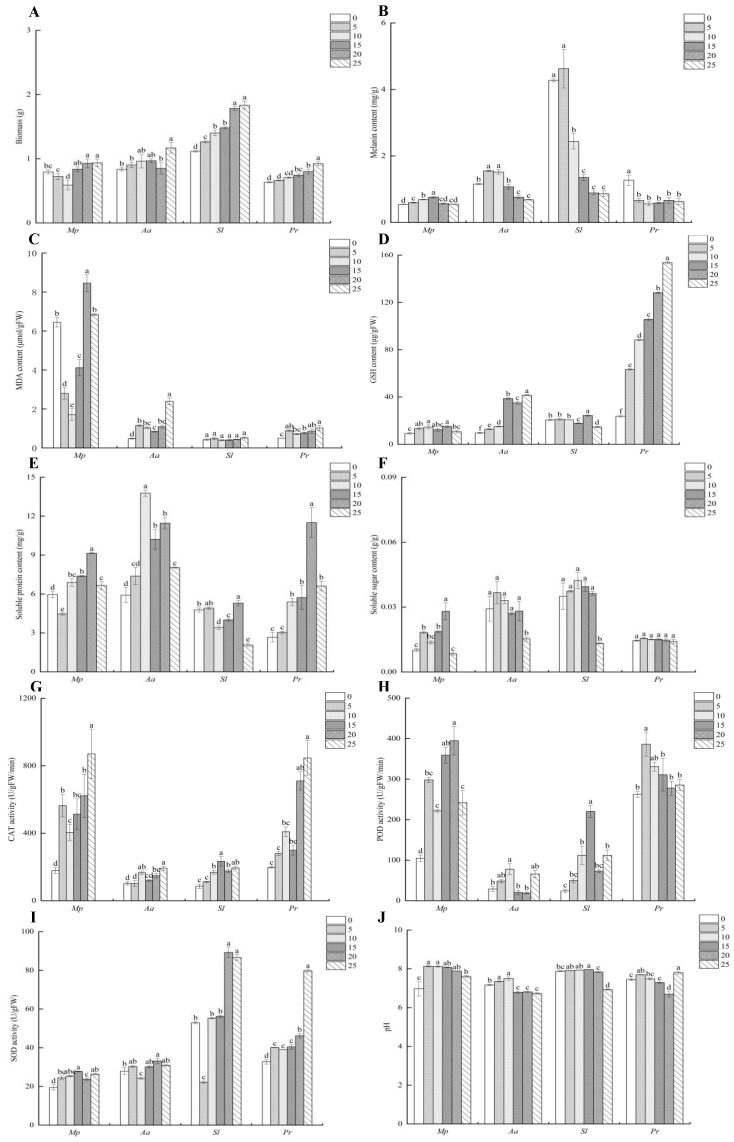
Growth and physiological indexes of DSE strains under liquid culture conditions. *Mp*, *Aa*, *Sl*, and *Pr* denote *M. pseudophaseolina*, *A. alstroemeriae*, *S. lupini*, and *P. radicina*, respectively. Biomass (**A**), melanin content (**B**), MDA content (**C**), GSH content (**D**), soluble protein content (**E**), soluble sugar content (**F**), CAT activity (**G**), POD activity (**H**), SOD activity (**I**), and pH (**J**) of DSE under drought stress. Different lowercase letters indicate significant differences between different DSEs (*p* < 0.05).

In the 25% drought treatment, the *Mp*, *Aa*, *Sl*, and *Pr* strains all had the most biomass, with values of 0.94, 1.17, 1.83, and 0.92 g, respectively (Figure 2A). The biomass of the *Sl* and *Pr* strains increased gradually as the stress level increased. The biomass in all stress treatments was significantly higher than that in the control treatment. Specifically, the biomass in the 25% drought treatment increased by 63.39% and 46.03% compared with the control. The biomass of *Mp* strains exhibited a fluctuating pattern, initially declining and then increasing, with a notable 18.99% increase after the 25% drought treatment compared with the control. The biomass of the *Aa* strain did not show a significant change under various drought stress conditions. However, the biomass during the 25% drought treatment was significantly greater than that of the control, with an increase of 40.96%.

The melanin levels in *Mp*, *Aa*, and *Sl* strains showed a trend of initially rising and then decreasing when exposed to increased stress levels compared with the control group (Figure 2B). The melanin concentration of the *Pr* strain did not show any notable variations after the different drought treatments. The melanin content of the *Mp* strain increased significantly with 5–15% drought stress compared with the control. It peaked at 15% drought stress, showing a 40.74% increase compared with the control. In the 5% drought treatment, the melanin content of the *Aa* and *Sl* strains achieved the maximum levels, showing a rise of 33.62% and 8.43%, respectively, compared with the control treatment.

The MDA content in the *Aa* strain significantly increased in all stress treatments compared with the control, peaking in the 25% drought treatment (Figure 2C). The MDA content of the *Pr* strain exhibited a substantial increase compared with the control in all stress conditions, except for the 10% drought treatment. As drought stress intensified, the MDA content of the *Mp* strain initially decreased and then increased, peaking in the 20% drought treatment. There was no notable variation in the MDA content of the *Sl* strain in all drought treatments when compared with the control treatment.

The GSH content of the *Aa* and *Pr* strains exhibited a substantial increase in comparison with the control under all drought stress regimens. The increase was gradual and reached a peak in the 25% drought treatment, showing a 329.64% and 541.83% increase, respectively, over the control (Figure 2D). In the 20% drought treatment, the GSH content of the *Mp* and *Sl* strains reached the highest levels, showing an increase of 72.22% and 18.65% compared with the control, respectively.

The soluble protein concentration of the *Mp*, *Sl*, and *Pr* strains peaked at 20% drought stress (Figure 2E). Compared with the control, the soluble protein content of the *Mp* and *Sl* strains in each drought treatment showed a decreasing and then increasing trend and reached its maximum value in the 20% drought treatment, which increased by 53.36% and 11.11%, respectively, compared with the control. The soluble protein content of the *Aa* and *Pr* strains gradually increased as drought stress intensified, peaking at 15% and 20%, respectively. The soluble protein content was 2.33 and 4.32 times greater than that of the control.

There was no notable variation in the soluble sugar concentration across the *Aa*, *Sl*, and *Pr* strains when exposed to drought stress (Figure 2F). The soluble sugar content of the *Mp* strain was markedly elevated compared with the control and other drought treatments at 20% drought, as it was three times that of the control.

When the drought stress worsened, the CAT activity of the *Mp*, *Aa*, *Sl*, and *Pr* strains went up compared with the control group (Figure 2G). At 25% drought stress, the CAT activity in *Mp*, *Aa*, and *Pr* strains peaked and rose by 386.60%, 87.5%, and 328.09%, respectively, compared with the control.

The POD activity of the *Mp*, *Sl*, and *Pr* strains rose when drought stress intensified compared with the control (Figure 2H). The POD activity of the *Aa* strain exhibited a pattern of initially rising, then falling, and then rising again as the drought stress intensified. In the 15% and 20% drought treatments, the POD activity of the *Sl* and *Mp* strains peaked and rose by 807.25% and 276.88%, respectively, compared with the control.

Both the *Mp* and *Pr* strains exhibited a notable increase in SOD activity under each drought stress condition compared with the control (Figure 2I). At 5–10% drought stress, the *Aa* and *Sl* strains exhibited a short-term decrease, whereas the SOD activity increased as the stress intensified.

The pH of each drought treatment of the *Mp* strain was significantly higher than that of the control, and the pH of each drought treatment was weakly alkaline (Figure 2J). The pH of the *Aa* strain initially rose and subsequently fell when exposed to prolonged dry conditions, transitioning from slightly alkaline to neutral, in contrast to the control group. The pH of the *Sl* strain did not show a significant difference compared with the control under any drought treatment; however, it tended to be neutral in the 25% drought treatment. The pH of each drought treatment of the *Pr* strain was consistently kept within the slightly alkaline range.

### 3.3. Determination of the Compensatory Properties of DSE Strains in Post-Drought Rehydration Treatments

**Figure 3:**
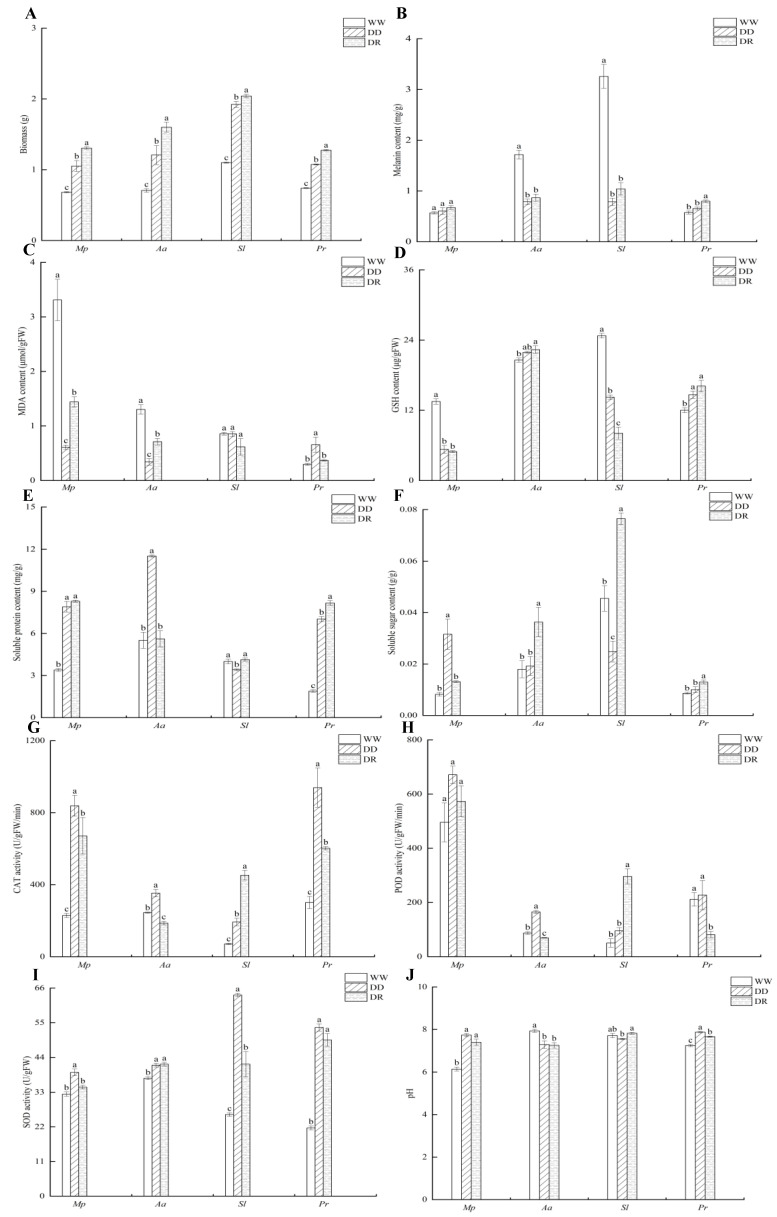
Growth and physiological indexes of DSE strains in drought and rehydration treatments. *Mp*, *Aa*, *Sl,* and *Pr* denote *M. pseudophaseolina*, *A. alstroemeriae*, *S. lupini*, and *P. radicina*, respectively. Biomass (**A**), melanin content (**B**), MDA content (**C**), GSH content (**D**), soluble protein content (**E**), soluble sugar content (**F**), CAT activity (**G**), POD activity (**H**), SOD activity (**I**), and pH (**J**) of DSE under drought and rehydration condition. WW, DD, and DR denote 0, 25% drought (before rehydration), and rehydration treatments, respectively. Different lowercase letters indicate significant differences between different DSEs (*p* < 0.05).

The biomass of the strains reached its maximum after rehydration and was significantly higher than that of the control and 25% drought treatment (before rehydration). The *Sl* strain showed the most significant biomass accumulation after rehydration, with biomasses of 1.31, 1.6, 2.04, and 1.28 g after rehydration in the *Mp*, *Aa*, *Sl*, and *Pr* strains, respectively (Figure 3A). The biomass of the *Mp*, *Aa*, *Sl*, and *Pr* strains increased by 92.65, 125.35, 85.45, and 72.97% after rehydration compared with the control. The biomass of the four DSE strains increased by 24.76, 32.23, 6.25, and 19.63%, respectively, after rehydration compared with that before rehydration.

Compared with the *Mp*, *Aa*, and *Pr* strains, the *Sl* strain accumulated the most melanin content after rehydration (Figure 3B). After rehydration, the melanin content of the *Pr* strain increased by 40.35% compared with the control and by 23.08% compared with that before rehydration. After rehydration, the melanin content of the *Mp*, *Aa*, and *Sl* strains increased somewhat compared with that before rehydration, but the difference was not statistically significant.

Compared with the control group, the MDA content of *Mp* and *Aa* strains did not increase before rehydration, while the MDA content of the *Pr* strain significantly increased (Figure 3C). Following rehydration, the MDA content returned to the control levels. The MDA concentration of the *Sl* strain did not change much before and after rehydration.

Compared with the *Mp*, *Sl*, and *Pr* strains, the *Aa* strain accumulated the most GSH content after rehydration (Figure 3D). Following rehydration, the GSH content in the *Aa* and *Pr* strains rose by 8.75% and 34.42%, respectively, compared with the control. The GSH content of the *MP* strain did not show a significant variation before or after rehydration.

The soluble protein level of the *Mp* and *Pr* strains was significantly higher before and after rehydration compared with that of the control treatment (Figure 3E). Post-rehydration, the soluble protein content of the *Sl* strain was much more significant than the pre-rehydration levels. The *Aa* strain exhibited elevated soluble protein levels before rehydration.

After rehydration, the soluble sugar content of the *Aa*, *Sl*, and *Pr* strains was significantly greater than both the control and the levels before rehydration (Figure 3F). The *Sl* strain accumulated the highest soluble sugar content after rehydration. After rehydration, the soluble sugar content of the *Aa*, *Sl*, and *Pr* strains increased by 101.66%, 68.13%, and 49.43% compared with the control. After rehydration, the soluble sugar content of the *Aa*, *Sl*, and *Pr* strains increased by 89.06%, 207.23%, and 30% compared with that before rehydration. While the soluble sugar content of the *Mp* strain rose after rehydration compared with that before rehydration, there was no significant difference in the changes in soluble sugar.

Compared with the *Aa*, *Sl*, and *Pr* strains, the *Mp* strain accumulated the most CAT activity after rehydration (Figure 3G). The CAT enzyme activity of the *Sl* strain peaked after rehydration, showing a 534% increase compared with the control and a 134.60% increase with respect to that before rehydration. After rehydration, the CAT activity of the *Mp* strain increased by 192.87%, and that of the *Pr* strain increased by 99.95% compared with the control.

Compared with the *Aa*, *Sl*, and *Pr* strains, the *Mp* strain accumulated the most POD activity after rehydration (Figure 3H). After rehydration, the POD activity of the *Sl* strain was much greater than both the control and the pre-rehydration levels; it increased by 484.31% and 209.73% in comparison with the control and pre-rehydration levels, respectively. The *Mp*, *Aa*, and *Pr* strains maintained elevated activity levels before rehydration, and POD activity did not return to normal after rehydration.

Compared with the *Mp*, *Aa*, and *Sl* strains, the *Pr* strain accumulated the most SOD activity after rehydration (Figure 3I). Following rehydration, the SOD activity of the *Aa*, *Sl*, and *Pr* strains increased by 11.72%, 61.52%, and 128.66%, respectively, compared with the control group.

After rehydration, the pH of the *Mp*, *Sl*, and *Pr* strains was higher than the control and leaned toward being slightly alkaline (Figure 3J). After rehydration, the pH of the *Sl* strain rose compared with that before rehydration, whereas there was no notable difference in the pH change after rehydration between the *Mp* and *Aa* strains.

### 3.4. Analysis of Variance Decomposition for Drought and Post-Drought Rehydration Treatments

**Figure 4:**
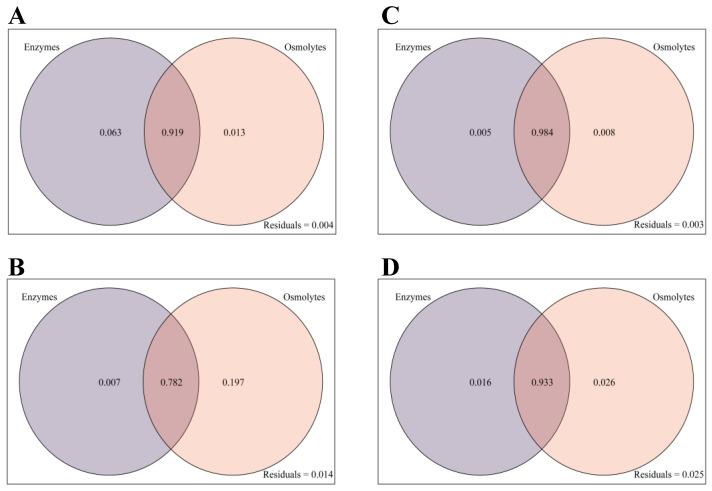
Variance decomposition plots of DSE under drought and rehydration treatments. (**A**–**D**) indicate *M. pseudophaseolina* (*Mp*), *A. alstroemeriae* (*Aa*), *S. lupini* (*Sl*), and *P. radicina* (*Pr*) strains, respectively. Enzyme means three antioxidant enzymes (CAT, POD, and SOD). Osmolytes represent three osmoregulatory substances (soluble protein, soluble sugar, and pH).

The contribution of different factors to the biomass of the *Mp*, *Aa*, *Sl*, and *Pr* strains was assessed through variance decomposition analysis (VPA) (Figure 4). The VPA results indicated that the combined explanation of antioxidant enzymes and osmoregulatory substances for the biomass of the *Mp* strain was 99.5%, with individual contributions of 6.3% and 1.3%, respectively, and an interaction effect of 91.9% (Figure 4A). For the *Aa* strain, the combined explanation was 98.6%, with individual explanations of 0.7% and 19.7% and an interaction effect of 78.2% (Figure 4B). In the case of the *Sl* strain, the combined explanation was 99.7%, with individual explanations of 0.5% and 0.8% and an interaction effect of 98.4% (Figure 4C). Lastly, for the *Pr* strain, the combined explanation was 97.5%, with individual explanations of 1.6% and 2.6% and an interaction effect of 93.3% (Figure 4D).

### 3.5. Metabolic Analysis of DSEs in the Drought and Rehydration Treatment

#### 3.5.1. Classification of PCA and Compounds Under Drought and Rehydration

**Figure 5:**
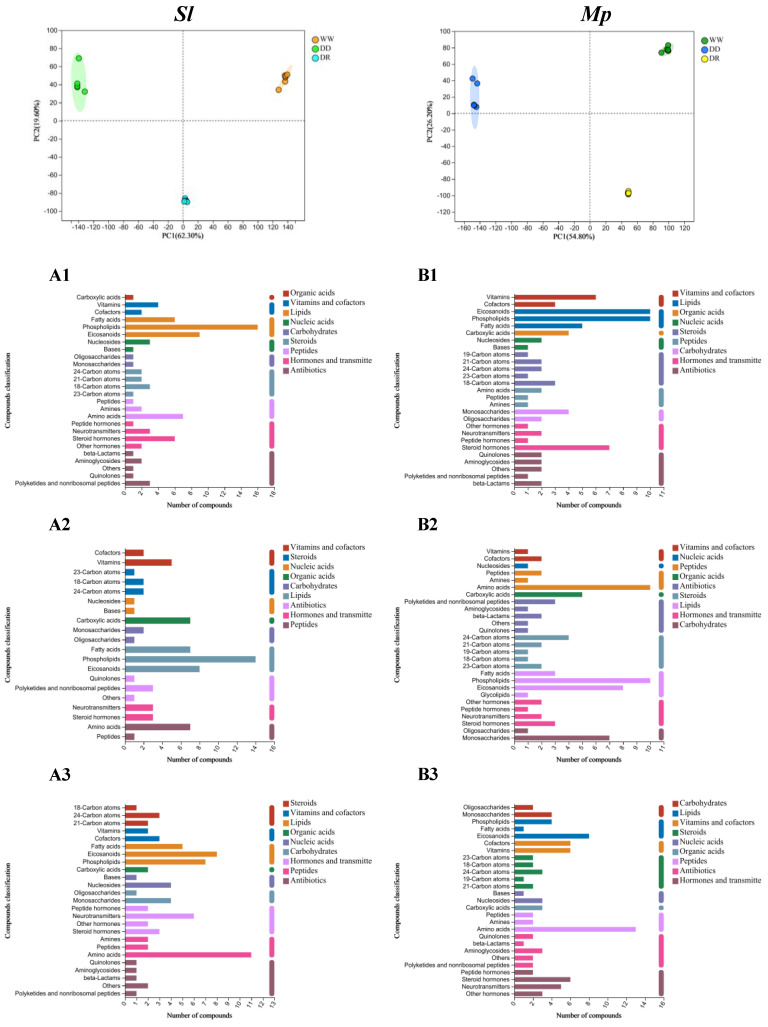
Plot of PCA scores and classification of compounds for different DSEs. (**A**,**B**) represent *S. lupini* (*Sl*) and *M. pseudophaseolina* (*Mp*) strains, respectively. WW, DD, and DR denote 0, 25% drought and rehydration treatments, respectively. Numbers 1–3 represented the changes of compounds in the three treatment groups of 25% vs. 0, rehydration vs. 0, and rehydration vs. 25%, respectively.

The strain growth rate and biomass were utilized as screening criteria. The *Mp* strain in the plate experiment reached the maximum diameter of plate growth just 4 d after experiencing 25% drought stress and rehydration. After rehydration in the liquid shake flask trials, the *Sl* strain exhibited the largest biomass increase of 85.45% compared with the control and 19.63% compared with the pre-rehydration time. The *Mp* strain showed a biomass increase of 92.65% compared with the control and 24.76% compared with the pre-rehydration period. Therefore, we selected the *Sl* and *Mp* strains in liquid culture for 21 d (0%, 25%, and rehydration) for subsequent metabolomic studies. PCA was utilized to monitor sample quality and assess the impacts of varying moisture conditions on the metabolites of the *Sl* and *Mp* strains throughout the drought and rewatering treatments. The first principal component of the *Sl* strain contributed 62.30%, whereas the second principal component contributed 19.60%. The first principal component of the *Mp* strain contributed 54.80%, while the second principal component contributed 26.20% (Figure 5). The samples of the two DSE strains were consistently grouped together under the same treatment circumstances, suggesting minimal variation within the group and demonstrating good repeatability and high data dependability. Distinct separation trends were seen among the various treatments, suggesting significant metabolite differences. The experimental outcomes were deemed satisfactory. Ultimately, the model was dependable.

Of the metabolites identified in all samples, 91 named metabolites, including organic acids, lipids, carbohydrates, nucleic acids, peptides, vitamins and cofactors, steroids, antibiotics, and hormone delivery mediator compounds, were found to be differentially expressed. Compared with the control group, the lipid and antibiotic compounds of the *Sl* strain in the 25% drought treatment (before rehydration) and after rehydration were significantly expressed (Figure 5(A1,A2)). Compared with before rehydration, the amino acid-related compounds induced by the *Sl* strain were significantly expressed following rehydration (Figure 5(A3)). Compared with the control group, the lipid and steroid compounds of the *Mp* strain were significantly expressed both before and after rehydration (Figure 5(B1,B2)). Compared with before rehydration, the amino acid-related compounds induced by the *Mp* strain were significantly expressed following rehydration (Figure 5(B3)). These results were consistent with the observation that lipid metabolism is an important way for plants to respond to growth and environmental stress.

#### 3.5.2. Statistics of Differential Metabolites Under Drought and Rehydration

**Figure 6:**
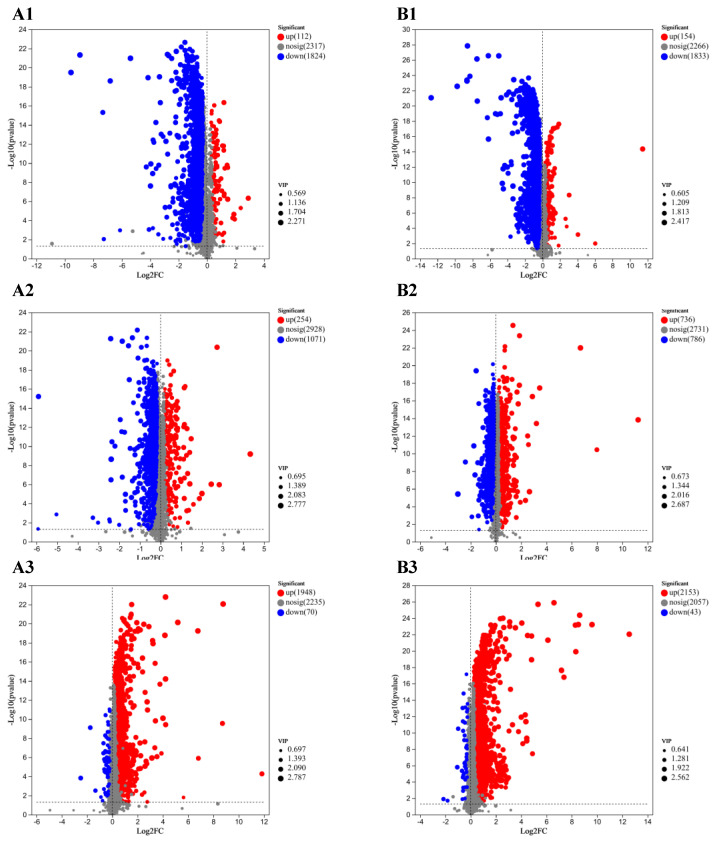
Volcano maps for different treatments of DSE. (**A**,**B**) represent *S. lupini (Sl)* and *M. pseudophaseolina (Mp)* strains, respectively. Numbers 1–3 represented the changes of compounds in the three treatment groups of 25% vs. 0, rehydration vs. 0, and rehydration vs. 25%, respectively.

This test took a combination of *t*-test and VIP values of OPLS-DA (*p* < 0.05 and VIP > 1) for differential metabolite screening (Figure 6). When compared with the control group, the *Sl* strain exhibited 1936 metabolites in the 25% drought treatment. Out of these, 1824 were downregulated and 112 were upregulated (Figure 6(A1)). The *Mp* strain had 1987 metabolites in the same treatment, with 1833 being downregulated and 154 being upregulated (Figure 6(B1)). The *Sl* strain had 1325 metabolites after rehydration, with 1071 downregulated and 254 upregulated metabolites compared with the control (Figure 6(A2)). The *Mp* strain had 1522 metabolites in the same treatment, with 786 being downregulated and 736 being upregulated (Figure 6(B2)). Compared with the 25% drought treatment, the *Sl* strain had 2018 metabolites after rehydration treatment, with 1948 upregulated and 70 downregulated metabolites (Figure 6(A3)). The *Mp* strain had 2196 metabolites in the same treatment, with 2153 upregulated and 43 downregulated metabolites (Figure 6(B3)). Overall, the *Sl* and *Mp* strains exhibited decreased and predominantly downregulated metabolite quantities during the 25% drought treatment and increased and predominantly upregulated metabolite quantities following rehydration.

#### 3.5.3. The Analysis of Metabolic Pathways of Differential Metabolites in the Drought and Rehydration Treatments

The different metabolites from each group were categorized into metabolic path-ways using the KEGG database to investigate the impacts of drought and rewatering treatments on DSE metabolic pathways. The screening of metabolic pathways at *p* < 0.05 and VIP > 1 showed that, in the *Sl* strain, a total of 78 metabolic pathways were annotated in the 25% vs. 0 treatment group, 82 pathways were annotated in the rehydration vs. 0 treatment group, and 83 metabolic pathways were annotated in the rehydration vs. 25% treatment group. For the *Mp* strain, 75 metabolic pathways were annotated in the 25 vs. 0 treatment, 80 pathways were annotated in the rehydration vs. 0 treatment, and 80 pathways were annotated in the rehydration vs. 25% treatment. These metabolic pathway functions were annotated as part of amino acid metabolism, lipid metabolism, cofactor and vitamin metabolism, bio-anabolism of other secondary metabolites, metabolism of terpenoids and polyketones, nucleotide metabolism, environmental response, and signal transduction metabolism.

**Figure 7:**
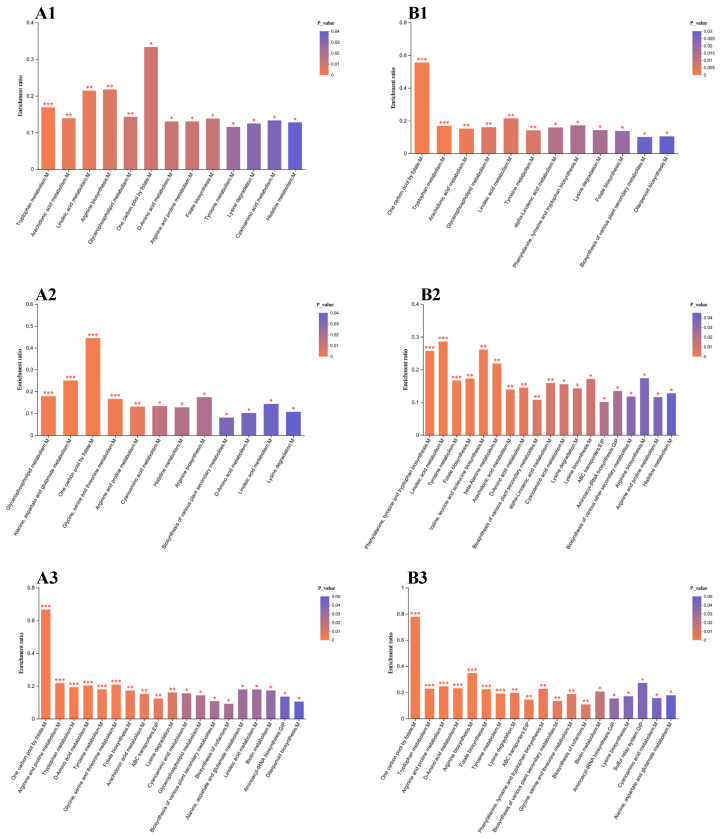
Plot of KEGG enrichment analysis for different DSEs. Significant metabolic pathways in different treatments of DSE. (**A**,**B**) represent *S. lupini* (*Sl*) and *M. pseudophaseolina* (*Mp*) strains, respectively. Numbers 1–3 represented the changes of compounds in the three treatment groups of 25% vs. 0, rehydration vs. 0, and rehydration vs. 25%, respectively. Horizontal coordinates in the graph indicate the pathway name and vertical coordinates indicate the enrichment rate. Color gradients of the bars indicate the significance of the enrichment, with darker colors representing a more significant enrichment of the pathway. *p*-value or FDR < 0.001 is marked as ***, *p*-value or FDR < 0.01 is marked as **, and *p*-value or FDR < 0.05 is marked as *.

The primary biological roles of certain metabolites were identified using KEGG pathway enrichment analysis in various treatments (Figure 7). Thirteen pathways were significantly enriched in the 25% drought-treated group of the *Sl* strain compared with the control, of which 14 differential metabolites were significantly upregulated in these pathways, mainly in lipid and amino acid metabolism, and the metabolites that were upregulated were CL(i-13:0/i-12:0/i-14:0/i-16:0) (CL1), PGP(18:1(9Z)/18:1(9Z)) (PGP1), PS(14:0/22:1(13Z)) (PS1), PA(18:4(6Z,9Z,12Z,15Z)/14:1(9Z)) (PA), N6-Acetyl-L-lysine, PS(22:4(7Z,10Z,13Z,16Z)/22:5(7Z,10Z,13Z,16Z,19Z)) (PS2), PS(18:2(9Z,12Z)/22:5(7Z,10Z,13Z,16Z,19Z))(PS3), CL(22:6(4Z,7Z,10Z,13Z,16Z,19Z)/18:1(9Z)/22:6(4Z,7Z,10Z,13Z,16Z,19Z)/20:4(5Z,8Z,11Z,14Z)) (CL2), PS(14:0/14:0) (PS4), CL(16:1(9Z)/22:5(4Z,7Z,10Z,13Z,16Z)/20:4(5Z,8Z,11Z,14Z)/22:5(7Z,10Z,13Z,16Z,19Z)) (CL3), CDP-DG, 8-amino-7-oxononanoic acid, methylimidazoleacetic acid, and phosphoglycolic acid. Most of these differential metabolites were enriched in the glycerophospholipid metabolic pathway, followed by the glycine, serine, and threonine, lysine degradation, and histidine metabolic pathways (Figure 7(A1)).

Twelve pathways were significantly enriched in the rehydration treatment of the *Sl* strain compared with the control, and 45 differential metabolites were significantly upregulated in these pathways. Among the metabolic pathways with a statistical significance of *p* < 0.001 (indicating high expression), particularly in lipids, amino acids, cofactors, vitamins, and metabolic pathways, 19 differential metabolites were identified as significantly upregulated. These upregulated metabolites included the following: 5-formiminotetrahydrofolic acid, aspartic acid, L-glutamic acid, CL1, PGP1, PS1, PE(18:4(6Z,9Z,12Z,15Z)/22:6(4Z,7Z,10Z,13Z,16Z,19Z)) (PE1), PE(20:5(5Z,8Z,11Z,14Z,17Z)/14:1(9Z)) (PE2), PA, PS2, PS3, CL2, LysoPC, PS4, CL3, CDP-DG, PGP(18:2(9Z,12Z)/18:2(9Z,12Z)) (PGP2), 4-acetamido-2-aminobutanoic acid, and dimethylglycine. These differential metabolites were enriched for glycerophospholipid metabolism, alanine, aspartate, and glutamate metabolism, glycine, serine, and threonine, and a metabolic pathway with highly significant expression of a folate carbon pool, respectively (Figure 7(A2)).

The *Sl* rewatering treatment had 19 significantly enriched pathways compared with the 25% drought treatment, and 231 differential metabolites were significantly upregulated in these pathways. Among the highly significantly expressed metabolic pathways, which were mainly amino acid, cofactor, and vitamin metabolic pathways, 47 differential metabolites were found to be significantly upregulated, and the upregulated metabolites were (6R)-folinic acid, folcidin, 5-formiminotetrahydrofolic acid, (6R)-5,10-methylenetetrahydrofolate, levomefolic acid, tetrahydrofolic acid, N-acetylputrescine, N(omega)-hydroxyarginine, octopine, D-octopine, trans-4-hydroxy-L-proline, sarcosine, creatine, N2-succinyl-L-ornithine, L-proline, guanidinoacetic acid, L-glutamate, 1-pyrroline-2-carboxylic acid, (3R,5S)-1-pyrroline-3-hydroxy-5-carboxylic acid, N2-succinyl-L-glutamic acid 5-semialdehyde, S-adenosylmethionine, subaphylline, 5-methoxyindoleacetate, quinoline-4,8-diol, 5-hydroxy-L-tryptophan, formyl-5-hydroxykynurenamine, 4-(2-aminophenyl)-2,4-dioxobutanoic acid, N’-formylkynurenine, 5-hydroxy-N-formylkynurenine, 5-methoxytryptamine, 6-hydroxymelatonin, 5-hydroxykynurenine, 2-amino-3-carboxymuconic acid semialdehyde, kynurenic acid, L-kynurenine, (E)-indol-3-ylacetaldoxime, 3,4-dihydroxyhydrocinnamic acid, tyramine, epinephrine, 5,6-dihydroxyindole-2-carboxylic acid, P-coumaric acid, maleic acid, vanillylmandelic acid, dopamine, maleylacetoacetic acid, salidroside, beta-tyrosine, 4-acetamido-2-aminobutanoic acid, PS3, 2-amino-3-methylsuccinic acid, dimethylglycine, aspartic acid, and APC. Most of these differential metabolites were enriched in the arginine and proline metabolism, tryptophan metabolism, D-amino acid metabolism, tyrosine metabolism, and glycine, serine, and threonine metabolism pathways, as well as, to a lesser extent, a metabolism pathway with highly significant expression of a folate carbon pool (Figure 7(A3)).

There were 12 metabolic pathways that showed significant differences in the *Mp* 25% drought-treated group compared with the control. Within these pathways, 15 differential metabolites were notably upregulated, and they were mainly concentrated in the biosynthesis of lipids, amino acids, and other secondary metabolites, as well as the metabolic pathways of terpenoids and polyketides. The upregulated metabolites were CL1, PGP1, PS1, PA, PS2, PS3, CL2, PS4, CL3, 3a,6b,7a,12a-tetrahydroxy-5b-cholanoic acid, N6-acetyl-L-lysine, saccharopine, coniferyl alcohol, esculetin, and sclareol, and most of these differential metabolites were enriched in the glycerophospholipid metabolic pathway, followed by phenylalanine, tyrosine, and tryptophan biosynthesis, biosynthesis of secondary metabolites, glycine, serine, and threonine metabolism, and lysine degradation in highly significantly expressed metabolic pathways (Figure 7(B1)).

The number of differentially significant pathways in the *Mp* rehydration-treated group compared with the control was 19. Among these pathways, 110 differential metabolites were significantly upregulated. Among the pathways with highly significant expression, which were mainly biosynthetic metabolic pathways of amino acids and other secondary metabolites, 21 differential metabolites were found to be significantly upregulated. The upregulated metabolites were chorismic acid, 3a,6b,7a,12a-tetrahydroxy-5b-cholanoic acid, L-tyrosine, L-quinate, 3-hydroxybenzoic acid, phenylalanine, epinephrine, leucodopachrome, tyrosol, dopamine, gentisic acid, 3,4-dihydroxyphenylpropanoate, 3-isopropylmalic acid, 2-isopropylmalic acid, L-isoleucine, L-valine, 4-aminobutyraldehyde, L-histidine, malonic acid, hydroxypropionic acid, and D-4′-phosphopantothenate. These differential metabolites were enriched in metabolic pathways for phenylalanine, tyrosine, and tryptophan biosynthesis and highly significant expression of tyrosine (Figure 7(B2)).

Nineteen pathways were significantly enriched in the *Mp* rewatering treatment compared with the 25% drought treatment, with 257 differential metabolites showing significant upregulation. Among the pathways with highly significant expression, which were mainly the biosynthesis of amino acids, cofactors and vitamins, other secondary metabolites, and lipid metabolism pathways, 76 differential metabolites were significantly upregulated. The upregulated differential metabolites were (6R)-folinic acid, folcidin, 5-formiminotetrahydrofolic acid, folic acid, (6R)-5, 10-methylenetetrahydrofolate, levomefolic acid, 7, 8-dihydrofolate, 5, 6, 7, 8-tetrahydrofolic acid, tetrahydrofolic acid, N-alpha-Acetyl-L-citrulline, N-acetyl-L-glutamic acid, citrulline, argininosuccinic acid, L-glutamine, N2-acetyl-L-ornithine, ornithine, N-acetylputrescine, N(omega)-hydroxyarginine, octopine, homocarnosine, D-octopine, 4-aminobutyraldehyde, trans-4-hydroxy-L-proline, sarcosine, creatine, L-aspartate-semialdehyde, N2-succinyl-L-ornithine, L-proline, guanidinoacetic acid, L-glutamate, L-pyrroline-2-carboxylic acid, S-adenosylmethionine, subaphylline, 5-methoxyindoleacetate, 8-methoxykynurenate, quinoline-4-8-diol, 5-hydroxyindoleacetylglycine, 2-aminomuconic acid semialdehyde, N-acetylserotonin, 3-methyldioxyindole, 5-hydroxyindoleacetic acid, formyl-5-hydroxykynurenamine, 4-(2-aminophenyl)-2, 4-dioxobutanoic acid, 3-hydroxyanthranilic acid, indolepyruvate, N’-formylkynurenine, 5-hydroxy-N-formylkynurenine, 4-(2-amino-3-hydroxyphenyl)-2, 4-dioxobutanoic acid, 6-hydroxymelatonin, 2-amino-3-carboxymuconic acid semialdehyde, kynurenic acid, (E)-indol-3-ylacetaldoxime, norepinephrine, 3, 4-dihydroxyhydrocinnamic acid, 4-hydroxyphenylacetylglutamic acid, tyramine, epinephrine, hydroxyphenylacetylglycine, 5, 6-dihydroxyindole-2-carboxylic acid, leucodopachrome, homovanillin, vanillylmandelic acid, tyrosol, dopamine, salidroside, 2-(4-hydroxyphenyl)ethanol, 3, 4-dihydroxymandelic acid, chorismic acid, 2-amino-6-[(1R, 2S)-1, 2, 3-trihydroxypropyl]-7, 8-dihydro-3H-pteridin-4-one, 6-lactoyltetrahydropterin, 4-amino-4-deoxychorismate, tetrahydrofolyl-[Glu](2), 1, 3, 5-Trihydroxybenzene, 6-carboxy-5, 6, 7, 8-tetrahydropterin, neopterin, and dihydrobiopterin. These differential metabolites were enriched in a metabolic pathway with highly expressed folate carbon pool metabolism, arginine biosynthesis, folate biosynthesis, tryptophan metabolism, arginine and proline metabolism, D-amino acid metabolism, and tyrosine metabolism (Figure 7(B3)).

In summary, under 25% drought stress, the main metabolic pathways of the *Sl* and *Mp* strains were lipid metabolism and amino acid metabolism. This suggests that there may be a link between these pathways and the DSE strains’ ability to survive drought. After rehydration, the metabolites and metabolic pathways increased in the *Sl* and *Mp* strains. The metabolism of amino acids, cofactors, and vitamins showed significant expression. Differential metabolites were significantly upregulated compared with the control and before rehydration. This indicates that the rehydration treatments induced significant expression of metabolites in the DSE strains, suggesting that rehydration tended to enhance stress tolerance and alleviate drought stress to some extent. These data support the idea that amino acids can function as antioxidants or osmoprotectants to mitigate damage in DSEs under drought stress. Additionally, cofactors and vitamins can activate high levels of metabolic pathways and, thus, ensure microbial activity.

### 3.6. Correlation Heatmap Analysis Between DSEs’ Resistance Indicators and Differential Metabolites

**Figure 8:**
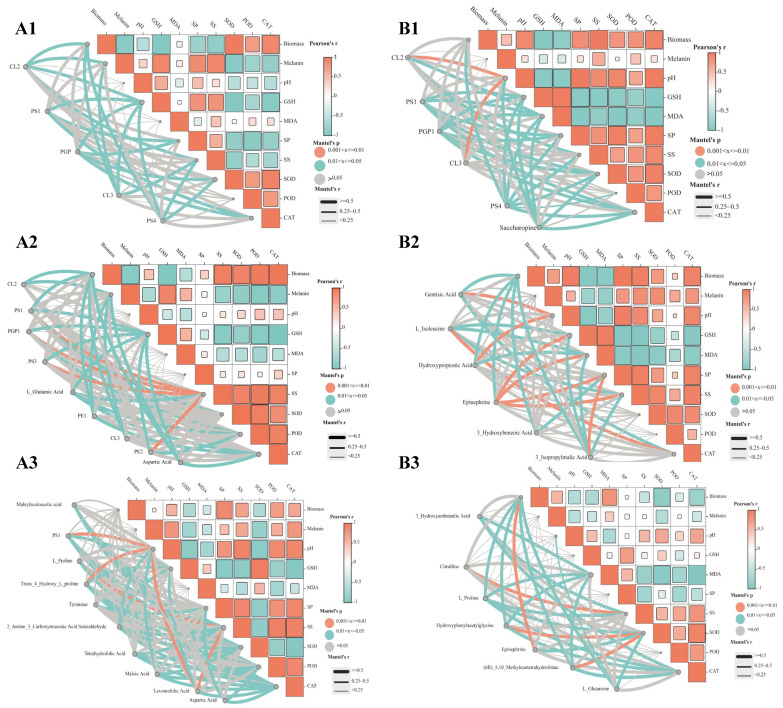
Mantel correlation heat maps between growth physiological indices and differential metabolites of DSE under different treatments. (**A**,**B**) represent *S. lupini* (*Sl*) and *M. pseudophaseolina* (*Mp*) strains, respectively. Numbers 1–3 represent the changes of metabolites in the three treatment groups of 25% vs. 0, rehydration vs. 0, and rehydration vs. 25%, respectively. The color gradient of the histogram indicates the significance of enrichment, and the darker the color, the more significant the enrichment. 0.001 < *p*-value ≤ 0.01 indicates highly significant, 0.01 < *p*-value ≤ 0.05 is marked as, and *p*-value > 0.05 is marked as not significant.

We analyzed the relationships among biomass, melanin, pH, GSH, MDA, soluble protein, soluble sugar, SOD, POD, CAT, and differential metabolites of the *Sl* and *Mp* strains (Figure 8). Compared with the control, the biomass, melanin, pH, GSH, MDA, soluble protein, soluble sugar, SOD, POD, and CAT of the *Sl* strain in the 25% drought treatment were significantly negatively correlated with the expression of CL2, CL3, PS1, PS4, and PGP metabolites (Figure 8(A1)).

Compared with the control group, the soluble sugar content of the *Sl* strain in the rewatering treatment was significantly positively correlated with the metabolites of PS1, PS3, PE2, and PGP1 (Figure 8(A2)).

Compared with the 25% drought treatment, the metabolite quantity of the *Sl* strain increased after rehydration. The soluble sugar content of the *Sl* strain was positively correlated with the metabolites of L_Proline and aspartic acid. pH was significantly positively correlated with the expression of PS3, aspartic acid, and trans_4hydroxy_L_proline metabolites (Figure 8(A3)).

Compared with the control, the pH of the *Mp* strain was significantly positively correlated with the expression of the CL2 and CL3 metabolites in the 25% drought treatment (Figure 8(B1)).

Compared with the control, the pH of the *Mp* strain was significantly positively correlated with the expression of gentisic acid and hydroxypropionic acid metabolites in the rehydration treatment. The expression of GSH, MDA, soluble protein, and soluble sugar was positively associated with epinephrine metabolites. soluble protein was positively correlated with the expression of 3_isopropylmalic acid metabolites. There was a significant positive correlation between CAT and L_isoleucine metabolite expression (Figure 8(B2)).

Compared with the 25% drought treatment, the metabolite quantity of the *Mp* strain increased after rehydration. The biomass of the *Mp* strain was positively correlated with the expression of epinephrine metabolites. The content of soluble sugar was positively correlated with the expression of citrulline metabolites. The SOD content and the hydroxyphenylacetylglycine and (6R)_5, 10_methylenetetrahydrofolate metabolites were very significantly positively correlated with the expression quantity (Figure 8(B3)).

### 3.7. Growth Parameters

**Figure 9:**
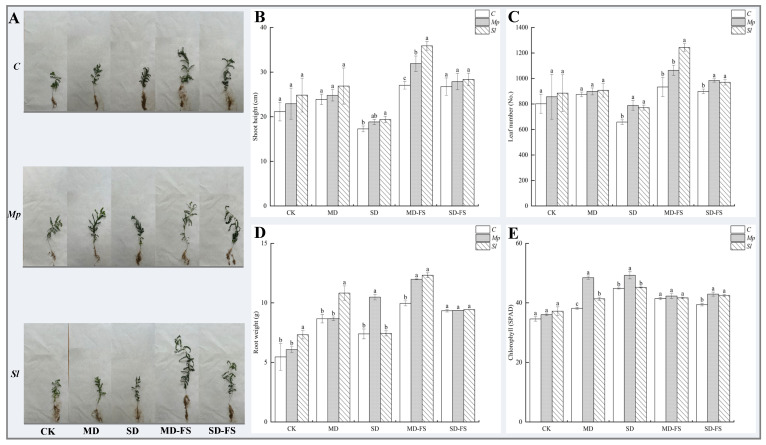
Effects of different DSE on growth morphological parameters of *Astragalus membranaceus* seedlings under drought and rehydration treatment. Growth diagram (**A**), plant height (**B**), leaf number (**C**), root weight (**D**), and chlorophyll (**E**) of *A. membranaceus* under drought and rehydration treatment. The abbreviations in the figure are a non-inoculated plant (*C*), *M. pseudophaseolina* (*Mp*) and *S. lupini* (*Sl*). The treatments CK, MD, SD, MD-FS, and SD-FS depicted in the figure represent normal water (70% of field water capacity), moderate drought (50% of field water capacity), severe drought (30% of field water capacity), post-medium drought replenishment, and post-severe drought replenishment, respectively. Means followed by the different letter(s) within each column significantly differ at *p* < 0.05.

The plant height, leaf count, and root fresh weight of plants following the rehydration treatment showed some compensatory effects when compared with those from before rehydration (Figure 9). The plant height, number of leaves, and root weight were better in the rehydration treatment after moderate drought (Figure 9A). The plant height, number of leaves, and root weight increased under all treatment groups inoculated with *Mp* and *Sl* strains compared to the uninoculated group. The greatest increase was observed in the *Sl* treatment. A significant increase of 32.75%, 33.24%, and 23.82% in plant height, number of leaves, and root weight, respectively, was observed in the *Sl*-inoculated group under rehydration after moderate drought compared with the uninoculated treatment (Figure 9B–D). The chlorophyll content of DSEs inoculated under moderate drought stress was significantly increased compared with that in the uninoculated treatment. Chlorophyll accumulation was most significant under severe drought stress (Figure 9E).

## 4. Discussion

### 4.1. Response of DSEs’ Growth Indicators to Drought and Severe Post-Drought Rehydration

DSEs can adapt effectively to drought conditions, as demonstrated by the experiments using PEG-6000 to simulate drought-stressed habitats. All strains except the *Pe* strain thrived well under varying degrees of drought stress [29]. Furthermore, studies revealed that different DSE strains displayed distinct growth characteristics; the *Sl*, *Mp*, and *Pr* strains reached the maximum plate growth diameter first at a 20% PEG-6000 concentration, while *Mc* had the highest growth rate at a 15% PEG-6000 concentration. These findings suggested that the growth characteristics of DSEs under drought conditions are closely related to the fungal strain [30]. Numerous studies have demonstrated that biomass is one of the most important metrics for assessing a plant’s ability to withstand drought and that drought stress significantly reduces biomass and inhibits plant development [31]. The current study shows that fungus rehydrates after suffering severe drought, and it is difficult to stimulate the compensatory effects of its growth and physiological indicators [8]. The results of this study showed that the biomass of the *Mp*, *Aa*, *Sl*, and *Pr* strains reached the maximum value under 25% drought stress, with the *Sl* strain accumulating the maximum biomass under 25% drought stress. After rehydration, the biomass of the *Mp*, *Aa*, *Sl*, and *Pr* strains was significantly higher than that of the control and that before rehydration. These results indicate that DSE strains have a significant positive effect on biomass under drought stress [32].

### 4.2. Response of the Physiological Indicators of DSEs to Drought Stress and Severe Post-Drought Rehydration

Drought stress induces oxidative stress, resulting in cellular damage, and MDA is commonly used as a crucial biomarker for evaluating drought resistance in plants [33]. Prior research demonstrated that elevated drought stress leads to a steady rise in MDA content [34]. This study demonstrated that the variations in the MDA content of the *Sl* strain did not exhibit statistically significant differences when the drought stress levels increased in comparison with the control group. The MDA content in the *Sl* strain decreased following rehydration in comparison with both the control and the pre-rehydration levels. The *Sl* strain might cope with drought stress by maintaining the health of its antioxidant system [35]. Under drought stress, the MDA content of the *Mp* strain exhibited a fluctuating pattern of decreasing and then increasing. Compared with the control group, the MDA content significantly decreased both before and after rehydration, with the most notable decrease occurring before rehydration. This decrease may have been due to the *Mp* strain restoring the MDA content to normal levels through self-repair mechanisms when exposed to severe drought stress [36]. Previous research indicated that plants with reduced MDA levels under drought circumstances have higher drought tolerance, indicating that the *Sl* and *Mp* strains are extremely resilient to drought stress. Compared with the control, the 25% drought treatment of the *Aa* strain significantly increased the MDA content at 14 d of stress but significantly decreased the MDA content at 21 d. This suggested that the *Aa* strain may have adapted to the drought environment with the increase in drought time, resulting in a decrease in MDA content. The MDA content in the *Pr* strain was greater under various drought conditions compared with the control treatment. Following rehydration, the MDA content of the *Pr* strain decreased considerably compared with that before rehydration and then gradually returned to an average level. This suggests that the *Pr* strain itself is less drought-resistant and that damage caused by drought stress can be mitigated by rehydration.

Melanin generated by DSEs during growth acts as an antioxidant, stimulates plant secretory enzymes, strengthens cell walls, and helps host plants absorb nutrients and water to survive harsh conditions [37]. This study demonstrated that the melanin content of the *Mp*, *Aa*, and *Sl* strains increased the most between the 5% and 15% drought treatments as the drought stress intensified. However, the melanin content of these strains did not increase under 20–25% drought stress. In contrast, the melanin content of the *Pr* strain did not increase under any level of drought treatment, indicating that melanin levels may vary among different fungi and can be influenced by the severity of drought stress [20]. There was no statistically significant difference in melanin content variations among the *Mp*, *Aa*, and *Sl* strains before and after rehydration, suggesting that the rehydration treatment did not stimulate melanin synthesis in the DSE strains.

Current studies have shown that SOD, CAT, and POD are the main antioxidant enzymes in living organisms because they eliminate ROSs produced when oxidation causes cell damage [38,39]. SOD and CAT activities of maize (*Zea mays* L.) increased under mild and moderate drought stress, but decreased with the intensification of water stress [40]. *Astragalus membranaceus* leaves exhibited a large increase in SOD, POD, and CAT activities under mild and moderate drought stress, but there was a considerable decrease in these activities under severe drought stress [41]. This study discovered that the *Mp*, *Aa*, *Sl*, and *Pr* strains collaborated with three enzymes to efficiently eliminate ROSs and prevent membrane lipid peroxidation in cells experiencing drought stress. The SOD activity of the *Mp* and *Pr* strains was significantly greater than the control in various drought treatments. The *Aa* and *Sl* strains exhibited significantly higher SOD activities under 20% drought conditions. Following rehydration, the levels of SOD in the *Mp*, *Aa*, *Sl*, and *Pr* strains were elevated compared with the control group. This suggested that DSE strains might eliminate ROSs by increasing SOD accumulation, and their ability to synthesize SOD is more readily triggered under intense drought conditions [42]. The CAT and POD activity of the *Mp*, *Aa*, *Sl*, and *Pr* strains exhibited varied increases in response to different drought treatments compared with the control. *Mp*, *Aa*, *Sl*, and *Pr* exhibited high POD activity to mitigate peroxidative damage despite a modest reduction in CAT activity. Following rehydration, the CAT and POD activity of the *Sl* strain dramatically increased compared with the control and pre-rehydration levels. This showed that the four DSE strains could use the three enzymes to effectively remove ROSs and had a high antioxidant capacity, especially when they were subjected to severe drought stress. GSH, a major enzyme in the non-enzymatic system, plays a crucial role in eliminating excess ROSs from organisms [43]. In this study, *Mp*, *Aa*, *Sl*, and *Pr* all peaked during 20–25% drought stress. After rehydration, the GSH content of the *Aa* and *Pr* strains increased, which was consistent with the previous finding that DSEs enhance the antioxidant activity of plants, leading to an increase in GSH content under heat-stress conditions [44]. However, the response of different DSE strains to GSH under drought conditions may differ.

Previous research indicated that DSE strains tend to accumulate more soluble protein under dry conditions [45]. In this study, it was found that the soluble protein content of the *Aa* strain increased under all drought stresses compared with the control. However, the soluble protein content after rehydration did not surpass that of the pre-rehydration treatment, suggesting that soluble protein is the primary factor influencing the *Aa* strain’s drought tolerance. The soluble protein contents of the *Mp*, *Sl*, and *Pr* strains exhibited a pattern of initially increasing and subsequently dropping in various drought treatments compared with the control. After rehydration, the soluble protein content of the *Mp*, *Sl*, and *Pr* strains increased compared with that before rehydration and the control. This suggests that the strains underwent rehydration to trigger a compensatory effect, enhancing the drought tolerance of DSEs. This improvement may be linked to the various strategies that DSEs employ to manage oxidative stress [34]. There was no significant difference in soluble sugar content among *Aa*, *Sl*, and *Pr* strains under various drought treatments. However, a reduction in soluble sugar content was noted in *Mp*, *Aa*, and *Sl* strains under severe drought stress. This decrease may have been due to the high levels of ROSs generated in the drought-stressed environment, which hindered soluble sugar production. This finding aligns with prior research; under drought stress, the content of soluble sugar in leaves decreased [46]. After rehydration, the soluble sugar contents of the *Mp*, *Aa*, *Sl*, and *Pr* strains returned to normal levels. Additionally, the soluble sugar contents of the *Aa*, *Sl*, and *Pr* strains were notably greater than those before rehydration, indicating a compensatory response in the soluble sugar contents of DSEs after rehydration.

### 4.3. Correlation Analysis of Physiological Indexes and Metabolism of DSEs Under Drought and Severe Post-Drought Rehydration

During drought stress, plants preserve the integrity of cellular structure by modifying the lipid content of cell membranes [47]. Studies have demonstrated that higher lipid accumulation levels can enhance plants’ tolerance to drought stress and improve plants’ resilience following rehydration [48]. This study found that glycerol phospholipid metabolites from the *Sl* and *Mp* strains accumulated significantly under 25% drought stress. CL2 and CL3 metabolites were notably expressed in the two DSE strains. This was consistent with current findings that lipid metabolism in arbuscular mycorrhizae plays a key role in tolerance to various abiotic stresses, such as drought [49]. Furthermore, drought stress can cause damage to cell membrane lipids, and plants can mitigate the harmful effects of ROSs by establishing an antioxidant system comprising SOD, CAT, and POD [50]. This study found a strong link between the *Sl* and *Mp* strains’ lipid metabolites and the SOD, CAT, and POD enzymes when they were treated with 25% drought. The above research results demonstrated that the DSE strains’ antioxidant defenses significantly increased in response to lipid peroxidation.

After rehydration, lipid-related metabolites significantly decreased in the *Sl* and *Mp* strains. The amino acid-related metabolites showed considerable expression, indicating that the metabolites of DSEs progressively recovered from drought stress after rehydration, which was possibly because of the efficient removal of ROSs by lipid metabolites working together with antioxidant enzymes. Previous findings have shown that whether plants survive drought depends on their ability to protect critical cellular components from drought stress and repair cellular biological structural functions under recovery conditions [51]. Amino acids at higher concentrations are believed to boost plant defense by influencing many physiological processes, such as controlling variations in osmotic pressure and acting as scavengers of ROSs [52]. Our results showed significant expression of amino acid, cofactor, and vitamin metabolism in the *Sl* and *Mp* strains after rehydration, as well as a significant increase in metabolites in the tryptophan, glycine, serine, threonine, tyrosine, arginine, and proline and folate carbon pool metabolic pathways. Similar findings were reported in studies involving soybeans and sesame [53,54]. After rehydration, the soluble sugar content of the *Sl* and *Mp* strains was significantly positively correlated with amino acid metabolites. This suggested that after rehydration, the osmotic pressure content and metabolites in vivo worked together to restore drought tolerance in the strains. This was consistent with previous research results showing that plants produce a large number of ROSs under drought stress. In order to maintain plant growth and development, plants induce the expression of amino acid-related metabolites in response to drought stress [17].

### 4.4. Effect of DSE on the Growth Performance of Astragalus Membranaceus Under Drought and Rehydration Conditions

DSEs widely colonize various ecosystems, especially under drought stress conditions, and DSE inoculation can improve plant resistance [55]. Current studies have shown that under the synergistic stress of drought and Cd, DSE inoculation can promote the growth of *A. membranaceus* [56]. Under drought stress, inoculation with *Arthrobacter endophyticus*, *Zobellella denitrificans*, and *Staphylococcus sciuri* in pistachio seedlings can significantly increase root weight and leaf number [57]. Another study showed that symbiosis with *P. tinctorius* favored the growth of cork oak under drought conditions, and mycorrhizal biomass increased under drought compared with non-mycorrhizal plants [58]. Our results showed that rehydration after moderate drought stress increased the plant height, leaf number, and root weight of *A. membranaceus*, but the compensation effects of different DSEs were different, which may have been due to the different effects of different DSEs on *A. membranaceus*. Compared with the severe drought treatment, the plant height and leaf number of all DSE-treated plants showed compensatory effects after rehydration after severe drought stress, indicating that DSE inoculation could effectively alleviate the damage caused by severe drought stress on *A. membranaceus* and had certain compensatory effects.

## 5. Conclusions

In this study, we found that the colony growth rates of *M. pseudophaseolina*, *S. lupini*, and *P. radicina* were the first to reach the maximum diameter for plate growth when the PEG concentration was 20%. With a PEG concentration of 25%, the *M. pseudophaseolina* strain reached the maximum diameter of the plate in as little as 4 d, which was about a week faster than the growth rate of the other strains. Under liquid culture conditions, the biomass of *S. lupini*, *M. pseudophaseolina*, *P. radicina*, and *A. alstroemeriae* strains increased gradually with increasing drought stress. After rehydration, the biomass of these strains peaked and was significantly higher than that of the control and 25% drought treatment (before rehydration). Therefore, the *S. lupini* and *M. pseudophaseolina* strains with drought tolerance and rehydration compensation were screened for subsequent differential metabolite analysis. It was further found that the essential metabolic pathways of the *S. lupini* and *M. pseudophaseolina* strains under drought stress were mainly lipid metabolism. After rehydration, the number of differential metabolites and metabolic pathways of the *S. lupini* and *M. pseudophaseolina* strains increased significantly, the content of lipid-related metabolites decreased in both strains, and most of the amino acid-related metabolites were expressed considerably, indicating that the metabolites of the two DSE strains were gradually restored after rehydration. In addition, rehydration after moderate drought stress significantly increased the aboveground index and root weight of *A. membranaceus*. Our research results, for the first time, screened out the functional DSE strains with both drought tolerance and rehydration compensation ability and verified the inoculation of *A. membranaceus* plants, thus providing a theoretical basis for the subsequent systematic study of microbial enhancers assisting medicinal plants in resisting severe drought to achieve the effect of water-saving cultivation of medicinal plants in arid areas.

## 6. Patents

A patent entitled “Application of a Dark Septate Endophyte of the Genus Chitococcus with Drought Tolerance and Rehydration Compensation Ability” is currently in the public phase. The patent number is CN202410286946.2.

## Figures and Tables

**Table 1:** Test DSE strains.

Strain	Host Plant	Geographic Location	Eco. Environment
*Stagonosporopsis lupini*	*Glycyrrhiza uralensis*	Chifeng in Inner Mongolia Autonomous Region	Farmland
*Microsphaeropsis cytisi*	*Glycyrrhiza uralensis*	Chifeng in Inner Mongolia Autonomous Region	Farmland
*Macrophomina pseudophaseolina*	*Astragalus membranaceus*	Anguo, Hebei Province	Farmland
*Paraphoma radicina*	*Astragalus membranaceus*	Anguo, Hebei Province	Farmland
*Alternaria alstroemeriae*	*Isatis indigotica*	Anguo, Hebei Province	Farmland
*Alternaria tellustris*	*Lycium ruthenicum*	Anxi, Gansu Province	Desert
*Papulaspora equi*	*Lycium ruthenicum*	Minqin, Gansu Province	Desert

**Table 2:** Experimental treatment scheme.

Groups	Methods
No drought stress group	70% field water capacity
Drought stress group	50% field water capacity 30% field water capacity
Rehydration group after drought	50% field capacity restored to 70% 30% field capacity restored to 70%
Inoculation group	*Mp* *Aa* *Sl*
Non-inoculation group	No DSE inoculation

## Data Availability

The original contributions presented in the study are included in the article/Supplementary Materials, further inquiries can be directed to the corresponding authors.

## Supplementary Materials

The following supporting information can be downloaded at: https://www.mdpi.com/article/10.3390/microorganisms12112254/s1, Table S1: Chromatographic and mass spectrometric parameters.
