# Supplementary material for: Characterization of Dark Septate Endophytes Under Drought and Rehydration and Their Compensatory Mechanisms in Astragalus membranaceus
Source: Microorganisms. 2024 Nov 7;12(11):2254. doi: 10.3390/microorganisms12112254 (PMC11596847; doi:10.3390/microorganisms12112254)
Supplement: Supplementary file 1 [file microorganisms-12-02254-s001.zip › microorganisms-3273981-supplementary.pdf]

Characterization of Dark Septate Endophytes Under Drought and Rehydration and Their Compensatory Mechanisms in *Astragalus membranaceus*

Yali Xie, Xueli He, Duo Wang, Menghui Wang, Wanyun Li, Wenjing Chen, Xianen Li and Chao He

Table S1. Chromatographic and mass spectrometric parameters.

	Description	Parameters
Mass Spectral Parameters	Scan type（m/z）	70-1050
	Sheath gas flow rate（arb）	50
	Aux gas flow rate（arb）	13
	Spray voltage（+）（V）	3500
	Spray voltage（-）（V）	-3500
	Heater temp（°C）	425
Chromatographic parameters	Column length × inner diameter × particle size of packing	100 mm×2.1 mm×1.8 μm
	Mobile phase A	95% water and 5% acetonitrile) (containing 0.1% (v/v) formic acid)
	Mobile phase B	(47.5%acetonitrile, 47.5% isopropanol and 5% water (containing 0.1% formic acid))
	Column temperature	40°C
	Injection volume	3 μL
